# First Report of *Bergeyella zoohelcum* Associated with Hemorrhagic Pneumonia in Forest Musk Deer (*Moschus berezovskii*): Evidence from Bacterial Culture, 16S rRNA Sequencing, and Metagenomic Analysis

**DOI:** 10.3390/microorganisms14071418

**Published:** 2026-06-29

**Authors:** Feiran Li, Lijuan Suo, Kun Bian, Kuo Sun, Chao Yang, Jie Tang

**Affiliations:** Shaanxi Key Laboratory of Qinling Ecological Security, Shaanxi Institute of Zoology, Xi’an 710032, China

**Keywords:** *Bergeyella zoohelcum*, hemorrhagic pneumonia, forest musk deer (*Moschus berezovskii*), bacterial culture, full-length 16S rRNA sequencing, viral metagenomics

## Abstract

Hemorrhagic pneumonia is a severe and often fatal disease in captive forest musk deer (*Moschus berezovskii*), but the pathogen remains incompletely understood. Based on incomplete statistics, the estimated incidence in captive populations ranges from 20% to 80%, with the disease occurring mainly in autumn, winter, and early spring. The disease has an acute onset and rapid progression. Due to the species’ strong stress response, affected animals rarely show behavioral changes, making early detection difficult. In this study, we investigated a mortality case presenting with oral bleeding and hematemesis on a forest musk deer farm. Postmortem examination revealed diffuse hemorrhagic pneumonia, and lung tissue samples were collected for histopathology, bacterial isolation, full-length 16S rRNA gene sequencing, and DNA/RNA virome sequencing. Histological examination showed extensive alveolar hemorrhage, fibrinous exudate, and macrophage infiltration. Bacterial culture and 16S rRNA gene sequencing identified *Bergeyella zoohelcum* as the predominant bacterium, accounting for 100% of the bacterial community in the lung tissue. Virome analysis revealed predominantly DNA bacteriophages (e.g., Cirlivirales, Cremevirales, Microviridae) and no known pathogenic RNA viruses; only seven low-abundance, unclassified RNA viral contigs of low completeness were detected. These results indicate that *B. zoohelcum* is the likely causative agent of hemorrhagic pneumonia in this case, with no evidence of viral involvement. This study provides the first direct association of *B. zoohelcum* with hemorrhagic pneumonia in forest musk deer, highlighting its pathogenic potential and the importance of monitoring this bacterium in captive populations.

## 1. Introduction

Hemorrhagic pneumonia is an acute and often fatal respiratory disease, frequently associated with bacterial, viral, or mixed infections [1,2]. In captive animals, such infections pose serious threats to both animal conservation and economic sustainability. The forest musk deer (FMD, *Moschus berezovskii*) is an endangered species highly valued for its medicinal musk; it is listed in Appendix II of CITES [3]. However, under captive breeding conditions, FMD is highly susceptible to respiratory diseases, which have currently become one of the primary causes of mortality in captive populations. Given the endangered status of FMD, each mortality event in captivity represents a significant loss for ex situ conservation efforts.

To date, *Escherichia coli* has been identified as one of the known causative pathogens of hemorrhagic pneumonia in forest musk deer [4], and other pathogenic bacteria such as *Streptococcus equines* [5] and *Klebsiella pneumoniae* [6] have also been reported to be associated with respiratory infections in this species. However, none of these studies reported the presence of *Bergeyella zoohelcum*. Despite their clinical and epidemiological importance, the overall pathogen spectrum underlying hemorrhagic pneumonia in captive forest musk deer remains incompletely elucidated, which severely restricts the ability to establish targeted diagnostic protocols and effective prevention and control strategies.

*Bergeyella zoohelcum* is a Gram-negative, aerobic, rod-shaped bacterium belonging to the family Flavobacteriaceae [7]. In veterinary medicine, *B. zoohelcum* has been isolated from the oral cavities of healthy dogs and cats [8], and it is also involved in respiratory diseases and systemic infections in a variety of animal hosts [9,10]. Notably, a recent study identified *B. zoohelcum* as the causative agent of hemorrhagic pneumonia in swine [11], further suggesting its emerging pathogenic potential in domestic and wild animals. Most reported cases of human infection by these bacteria are due to animal bites or are associated with prolonged exposure to pets [12,13]. Moreover, this pathogen can also cause bacteremia in immunocompromised populations and children with aanimal exposure [14,15].

Despite mounting evidence that *B. zoohelcum* causes respiratory and systemic infections in various animals, its association with hemorrhagic pneumonia in forest musk deer has not been documented. In this study, we investigate the presence of *B. zoohelcum* in forest musk deer with hemorrhagic pneumonia and characterize its clinical, pathological, and molecular features. Our findings provide the first observation of *B. zoohelcum* as a novel causative agent of hemorrhagic pneumonia in this endangered species, thereby expanding the spectrum of pathogens for fatal respiratory disease in captive forest musk deer and offering a scientific basis for clinical diagnosis, targeted prevention, and epidemiological control.

## 2. Materials and Methods

### 2.1. Case History and Sample Collection

The case involved a 9-month-old male forest musk deer from a captive farm. It was housed in an enclosure with an indoor shelter (3 m^2^) and an outdoor yard (15 m^2^). The outdoor yard was also shared by two other forest musk deer, with no other species present. The diet included fresh leaves (mulberry, elm). Water was supplied as cooled, boiled water. The enclosure floors were fully concreted with no access to vegetation.

Approximately one hour prior to death, the forest musk deer exhibited normal behavior, including regular feeding, drinking, and locomotor activity (running and jumping) within the enclosure. However, it abruptly developed oral hemorrhage and hematemesis. The carcass was found immediately after death within 30 min. A gross postmortem examination was performed within 2 h (the ambient temperature at the time of death was 4 °C), and lesioned lung tissue samples were then collected aseptically. The collected tissues were divided into three portions. The first portion was immediately placed on dry ice and subsequently transferred to a −80 °C freezer for virome and full-length 16S rRNA gene sequencing. The second portion was stored in sterile Eppendorf tubes (Eppendorf AG, Hamburg, Germany) at 4 °C for subsequent bacterial isolation and culture. The third portion, comprising tissues with obvious lesions, was fixed in 4% paraformaldehyde for histopathological examination.

### 2.2. Bacterial Isolation and Identification from Lung Tissue

Lung tissue was aseptically collected and used to prepare a 10^−1^ homogenate, which was then serially diluted and spread onto plates of routine nutrient agar, blood agar, chocolate agar, and MacConkey agar. The plates were incubated at 37 °C in a 5% CO_2_ incubator for 24–48 h. Based on differences in colony size and morphology, single colonies were selected and purified. The isolates were preliminarily screened by Gram staining and microscopy. Bacterial DNA was extracted using a bacterial genomic DNA extraction kit (Tiangen, Beijing, China) according to the manufacturer’s instructions. PCR amplification of the bacterial 16S rRNA gene was performed using universal primers [16]. The PCR reaction system and amplification program were as described in the literature [11]. The amplified products were submitted to Tsingke Biotechnology Co., Ltd. (Beijing, China) for sequencing, and species identification was conducted by combining colony morphology, staining characteristics, and BLASTn homology analysis (≥99%) against the NCBI GenBank database.

The phylogenetic tree was constructed using the Neighbor-Joining (NJ) method based on 16S rRNA gene sequences. Bootstrap analysis was performed with 1000 replicates to assess the reliability of the tree topology. All analyses were conducted using MEGA software (v 7.0) [17]. The tree was built by comparing the 16S rRNA sequences of the isolates with those of closely related species within the family Flavobacteriaceae, which were retrieved from the GenBank database.

### 2.3. Viral Enrichment Library Preparation and Next-Generation Sequencing

All experimental procedures, including sample pre-treatment, nucleic acid extraction, library construction, and sequencing, were performed by Guangdong Magigene Biotechnology Co., Ltd. (Guangzhou, China), following the methods described previously [18]. Briefly, viral-like particles (VLPs) were enriched from *Moschus berezovskii* lung tissues by grinding, freeze–thaw cycling, low-speed centrifugation, serial filtration, and ultracentrifugation through a 28% sucrose cushion at 160,000× *g* for 2 h at 4 °C using a HIMAC CP 100WX ultracentrifuge (Hitachi, Tokyo, Japan). Viral nucleic acids were extracted using the TaKaRa MiniBEST Viral RNA/DNA Extraction Kit Ver.5.0 (Takara, Japan). The quality of the amplified products was then assessed using Thermo NanoDrop One (Thermo Fisher Scientific, Waltham, MA, USA), Life Technologies Qubit 4.0 (Thermo Fisher Scientific, USA) for quantitation, and 1.5% agarose gel electrophoresis for integrity evaluation. Libraries were constructed using the ALFA-SEQ DNA Library Prep Kit (Findrop Biosafety Technology (Guangzhou) Co. Ltd., Guangzhou, China) following the manufacturer’s protocol. Sequencing was performed on an Illumina Novaseq 6000 with paired-end 150 bp (PE150).

### 2.4. Sequence Analysis and Virus Identification

Raw sequencing reads were quality-filtered using Trimmomatic [19] (v 0.40) to remove adapter sequences, PCR duplicates, and polyX sequences. Reads containing >20% low-quality bases (Phred score ≤ 20) were discarded to obtain high-quality clean reads. Subsequently, clean reads were aligned against the host (*Moschus berezovskii*, assembly ASM2237691v1, RefSeq: GCF_022376915.1) and ribosome (Silva v132) databases using BWA [20] (v0.7.17). Alignments with a length less than 80% of the total read length were filtered out. The high-quality reads were assembled using MEGAHIT [21] software (v 1.2.9). Assembled contigs were compared against the host genome using BLAST v2.9.0+.

Potential viral contigs were identified using CheckV [22] (v0.8.1) and Virsorter2 [23] (v2.2.3). Briefly, CheckV identified host contamination and prophages via hidden Markov models (HMM) [24], followed by amino acid identity (AAI) and HMM analyses to assess sequence confidence and completeness. High-completeness sequences (>90%) were further evaluated for direct terminal repeats (DTR) or inverted terminal repeats (ITR) to predict complete viral structures. Concurrently, Virsorter2 identified viral sequences based on gene content and genomic features to corroborate CheckV results, and its high-confidence outputs were retained as complements. Viral taxonomic annotation was performed using the PhaGCN2 [25] software (v2.0).

### 2.5. Full-Length 16S rRNA Sequencing

Sample DNA extraction was performed by Biomarker (Beijing Biomarker Technologies Co., Ltd., Beijing, China) using TGuide S96 Magnetic Soil/Stool DNA Kit (Tiangen Biotech (Beijing) Co., Ltd., Beijing, China) according to the manufacturer’s instructions. DNA concentration and purity were assessed using Qubit 3.0 (Thermo Fisher Scientific, USA) and NanoDrop One (Thermo Fisher Scientific, USA). The full-length bacterial 16S rRNA gene was amplified by PCR using the barcoded primers (27F: AGRGTTTGATYNTGGCTCAG; 1492R: TASGGHTACCTTGTTASGACTT). The PCR products were sequenced on the PacBio Sequel II platform (Illumina, San Diego, CA, USA), and library preparation was performed following the 16S Amplification SMRTbell^®^ Library Preparation standard protocol (PacBio). Raw sequencing data in BAM format were demultiplexed using Lima software (v2.7.1). Operational taxonomic units (OTUs) were clustered using UPARSE (v10.0) [26]. Representative sequences were aligned against the SILVA v138.1 database [27] for taxonomic annotation using the QIIME2 feature-classifier classify-consensus-blast (default confidence threshold of 0.8).

## 3. Results

### 3.1. Gross and Histopathological Features of Hemorrhagic Pneumonia in a Forest Musk Deer

**Figure 1:**
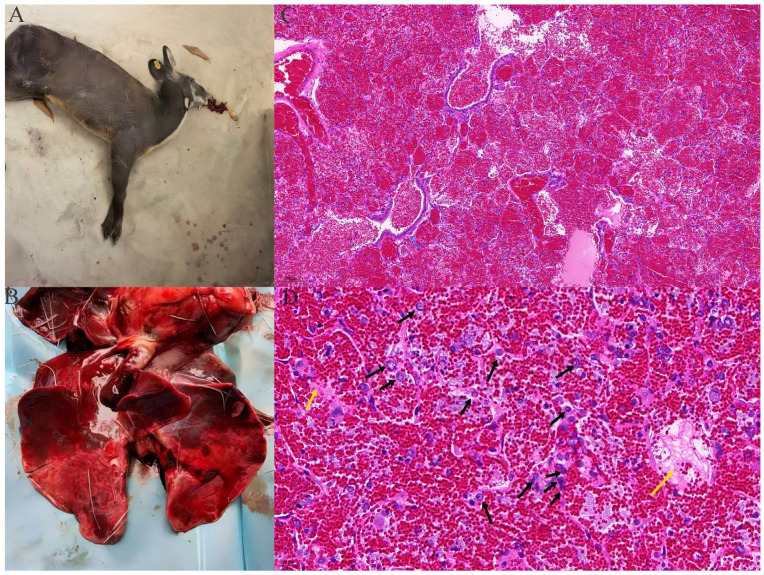
Gross (**A**) and histopathological (**B**) findings in the lungs of a deceased forest musk deer. (**A**) Appearance of the dead forest musk deer, with blood visible at the nostrils and mouth. (**B**) Gross lesions of the affected lung, showing diffuse dark red discoloration and a moist cut surface. (**C**) H&E staining (10×): Diffuse pulmonary hemorrhage, with numerous red blood cells filling the alveolar spaces and bronchioles (Blue arrow). (**D**) H&E staining (40×): fibrinous exudate (yellow arrow) in the alveolar spaces, with infiltrating macrophages indicated by black arrows.

Gross pathological examination of the dead forest musk deer revealed bloody discharge from the nostrils and mouth (Figure 1A). At necropsy, approximately 85% of the lung surface showed diffuse dark red discoloration with a moist, oozing cut surface, indicative of severe congestion and hemorrhage (Figure 1B). Histopathologically, the lung tissue showed diffuse hemorrhagic lesions. The alveolar spaces and bronchioles were diffusely filled with numerous erythrocytes, obscuring normal alveolar architecture (Figure 1C). The alveolar lumina contained abundant erythrocytes, and fibrinous exudate was observed in some alveolar spaces, accompanied by macrophage infiltration (Figure 1D).

### 3.2. Bacterial Culture and Identification

**Figure 2:**
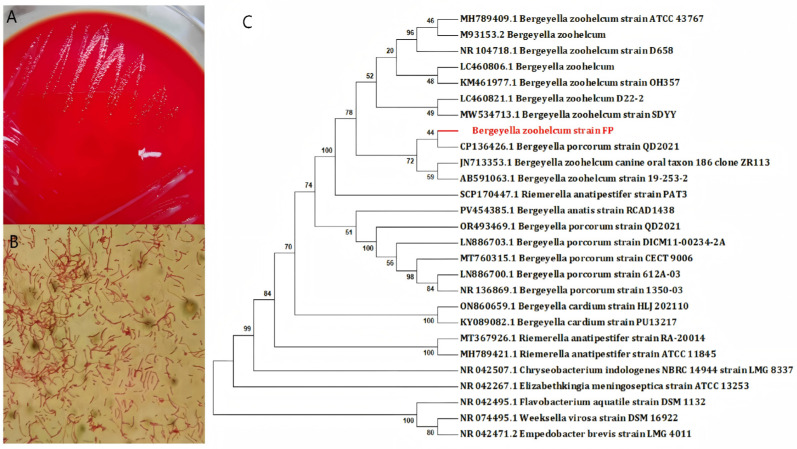
Morphological and molecular identification of the isolated bacterial strain *B. zoohelcum.* (**A**) Colony morphology of the isolate cultured on blood agar plates at 37 °C for 48 h, showing small, circular, non-hemolytic colonies. (**B**) Gram staining of the isolated strain, revealing Gram-negative, rod-shaped bacteria under light microscopy (40× magnification). (**C**) Phylogenetic tree based on the 16S rRNA gene sequences of the isolated strain and related reference strains. The tree was constructed using the neighbor-joining method in MEGA software, with bootstrap values (1000 replicates) shown at the nodes. The isolated strain (marked in red) clustered within *Bergeyella zoohelcum*, confirming its taxonomic identity.

Colonies grown on blood agar after 48 h incubation at 37 °C under 5% CO_2_ were small, punctate, translucent, and smooth-edged (Figure 2A), revealing Gram-negative rods with rounded ends and parallel sides (Figure 2B). The 16S rRNA sequences obtained were compared using the NCBI database. The obtained sequences showed a high similarity (99.86%) with the *B. zoohelcum canine oral taxon 186 clone ZR113* (JN713353.1) and *Bergeyella zoohelcum 18-1 gene* (LC460812.1). Our isolates were preliminarily identified as *B. zoohelcum*, and the isolate was designated as strain *Bergeyella zoohelcum FP*.

The phylogenetic analysis showed that the isolate in this study (marked in red) clustered within the same clade as the reference strains of *Bergeyella zoohelcum* (Figure 2C), indicating its closest phylogenetic relationship with *Bergeyella zoohelcum*. Notably, strain FP clustered most closely with *Bergeyella porcorum strain QD2021* (CP136426.1) and formed a distinct subclade with other *Bergeyella zoohelcum* strains. In contrast, strain FP was clearly separated from *Bergeyella cardium* and related genera (*Riemerella*, *Chryseobacterium*, *Elizabethkingia*, etc.). The phylogenetic analysis confirmed that strain FP was identified as *Bergeyella zoohelcum*.

### 3.3. Identification of Bergeyella zoohelcum by Full-Length 16S rRNA Sequencing

A total of 28,363 circular consensus sequences (CCS) were obtained from the two samples after barcode identification, with each sample yielding at least 13,487 CCS (average 14,182 CCS). A sequencing quality assessment (Supplementary Table S1) indicated that the base quality, sequence length, and effective read counts met the requirements for microbial community analysis, confirming the reliability of the sequencing data for subsequent species annotation and community structure analysis.

**Figure 3:**
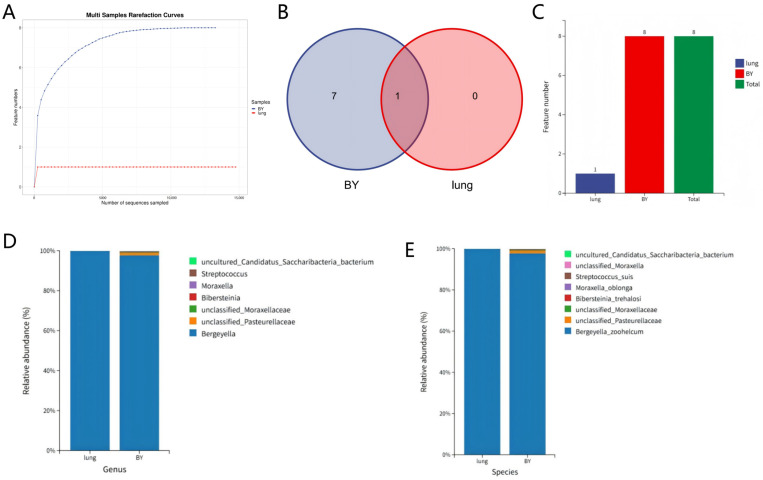
Bacterial diversity and composition analysis of lung and nasopharyngeal (BY) samples from the forest musk deer. (**A**) Rarefaction curves. The horizontal axis represents the number of sequence samples; the vertical axis represents the number of features. (**B**) Venn diagram showing the overlap of OTUs between the lung and nasopharyngeal samples. (**C**) Bar chart displaying the number of observed features in the lung, nasopharyngeal (BY), and combined (Total) datasets. (**D**) Relative abundance of bacterial communities at the genus level. (**E**) Relative abundance of bacterial communities at the species level.

Rarefaction curves constructed at the OTU level (Figure 3A) showed that the curves rose rapidly and then gradually plateaued as sequencing depth increased, indicating that the sequencing depth was sufficient to cover the majority of microbial species in the samples. Using a 97% sequence similarity threshold for OTU clustering, a total of eight bacterial OTUs were obtained (Figure 3B). Venn diagram analysis revealed that only one OTU was shared between the two samples (Figure 3C).

The taxonomic annotation of the representative OTU sequences classified all reads within the domain Bacteria, comprising four phyla, four classes, five orders, five families, seven genera, and eight species. At the genus level (Figure 3D), *Bergeyella* dominated the lung tissue samples, representing 100% of the reads. In the nasopharyngeal (BY) swabs, *Bergeyella* was also the predominant genus (97.86%), with minor contributions from unclassified_Pasteurellaceae (1.17%), unclassified_Moraxellaceae (0.54%), and Bibersteinia (0.29%). Species-level analysis showed that *B. zoohelcum* accounted for 100% of the bacterial community (Figure 3E), identifying it as the core dominant species within the lung tissue microbiota of the deceased forest musk deer.

### 3.4. Virome Sequencing and Assembly Results

In this study, DNA and RNA viromes of forest musk deer lung tissue samples were sequenced using Illumina paired-end high-throughput sequencing. Raw reads were subjected to quality control, adapter trimming, and removal of low-quality sequences using Trimmomatic V4.0 software to obtain high-quality clean reads. Detailed data processing statistics are presented in Supplementary Tables S2 and S3.

For the DNA virome (Supplementary Table S2), the raw data yielded 12.2 Gb (40,583,194 paired-end reads). After quality control, 8.3 Gb of high-quality data (27,656,817 clean reads) were obtained. Following host sequence filtration, 14,228,204 non-host reads (51.45% clean reads) were retained (Supplementary Table S2). De novo assembly of these non-host reads generated 30,559 DNA viral contigs, with a total base count of 34.7 Mb and an N50 of 1667 bp (Table 1).

For the RNA virome (Supplementary Table S3), the raw data yielded 9.8 Gb (32,717,452 paired-end reads). After quality control, 6.0 Gb of high-quality data (24,119,669 clean reads) were obtained. Following further removal of rRNA reads and host reads, 8,456,244 non-host reads (35.06% clean reads) were obtained (Supplementary Table S3). De novo assembly of these reads using MEGAHIT v1.2.9 software yielded 3563 contigs. After removing six host-derived contigs, a total of 3557 RNA viral assembly contigs were retained, with a total base count of 1.66 Mb and an N50 of 461 bp (Table 1).

Both datasets yielded sufficient high-quality virome reads after quality control and contaminant removal, meeting the requirements for subsequent viral sequence assembly and annotation.

### 3.5. Identification and Taxonomic Annotation of Viral Sequences

Based on DNA virome sequencing, a total of 1738 viral contigs (including 12 proviral sequences) were obtained (Table 2). Among these, 98 sequences exhibited complete genomic structures, consisting of 75 contigs with terminal repeat structures (DTRs) and 23 contigs identified using the AAI method. In terms of genome type, dsDNA viruses dominated, accounting for 66.51% (1156 contigs) of the total, followed by ssDNA viruses at 19.91% (346 contigs), whereas the remaining sequences (13.58%) were of undetermined genome types (Table 3). Bacteriophages showed a clear predominance in the viral community, representing 73.42% of all viral types and accounting for 75.85% of the relative abundance.

**Figure 4:**
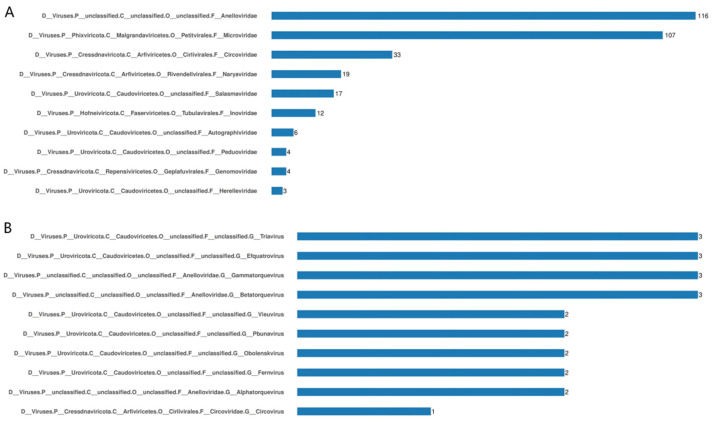
Viral community composition in lung tissue. (**A**) Taxonomic distribution of viral contigs at the family level. The top 10 most abundant families are shown, ranked by the number of contigs assigned to each family. (**B**) Viral community composition at the genus level, with contig counts for each genus.

Classification statistics showed that all 1738 viral contigs were annotated at the domain level, with 1506 contigs annotated at the phylum level, 1491 contigs at the class level, 405 contigs at the order level, 352 contigs at the family level, and 26 contigs at the genus level. At the family level (Figure 4A), Anelloviridae was the most abundant (116 contigs), followed by Microviridae (107 contigs), Circoviridae (33 contigs), Naryaviridae (19 contigs), and Salasmaviridae (17 contigs). Additional families detected included Inoviridae, Autographiviridae, Peduoviridae, Genomoviridae, and Herelleviridae. At the genus level, annotations were assigned to phage genera such as *Triavirus*, *Efquatrovirus*, *Vieuvirus*, *Pbunavirus*, *Obolenskvirus*, and *Fernvirus*, as well as viral genera including *Gammatorquevirus*, *Betatorquevirus*, *Alphatorquevirus* (all belonging to Anelloviridae), and *Circovirus* (belonging to Circoviridae). Most of these genera contained only 1–3 contigs (Figure 4B).

In contrast, only seven RNA viral contigs were identified, with a total length of 5233 bp (Table 2). All seven sequences were of low completeness and single-segment, and none could be assigned to a genome type or classified at any taxonomic level (e.g., family or genus). No known pathogenic RNA viruses were detected.

## 4. Discussion

This study reports a fatal case of hemorrhagic pneumonia in a forest musk deer. Using a multi-technology approach integrating histopathology, bacterial culture, full-length 16S rRNA gene sequencing, and metavirome analysis, *Bergeyella zoohelcum* was identified as the putative pathogen responsible for the disease. To the best of our knowledge, this is the first report of *B. zoohelcum* infection in forest musk deer, thereby extending both the known host range and the associated clinical disease spectrum of this bacterium. These findings not only enrich the natural host spectrum of *B. zoohelcum* but also provide key evidence for the etiological diagnosis of bacterial pneumonia in forest musk deer farming, with significant implications for veterinary clinical practice and wildlife conservation.

The pathological manifestations of hemorrhagic pneumonia in forest musk deer vary significantly depending on etiology. *Escherichia coli* has been isolated from hemorrhagic pneumonia in this host, with gross pathological findings of pulmonary congestion, hemorrhage, and pleural effusion or hemothorax, and the isolates were shown to be multidrug-resistant and pathogenic in mice [4]. *Streptococcus equinus* possesses a beta-hemolysin genomic island containing 12 cyl genes. Pathologically, it is manifested as thickening of the alveolar walls [5], while *Klebsiella pneumoniae* pathogenicity is regulated by complex virulence networks such as the LysR family gene kp05372 [6]. In contrast, the present case caused by *Bergeyella zoohelcum* was histopathologically characterised by marked alveolar hemorrhage with macrophagic and fibrinous exudation, along with 100% relative abundance of this pathogen in the lung microbiome—a finding that warrants further microbiological contextualization.

The isolation of *B. zoohelcum* from the lesioned lung tissue, together with the full-length 16S rRNA sequencing results, provides strong microbiological evidence linking the pathogen to the observed pathology. A comparative analysis of the bacterial community between the lung tissue and nasopharyngeal swabs revealed a striking contrast: while *Bergeyella* dominated both sites, the lung tissue exhibited near-complete monopolization by this genus, whereas the nasopharynx retained a more diverse microbiota including members of the families Pasteurellaceae, Moraxellaceae, and the genus *Bibersteinia*. In severe acute bacterial pneumonia, the lung microenvironment undergoes a drastic shift, often leading to the overgrowth of a single pathogenic species. This has been extensively documented for various respiratory pathogens, where the causative agent can overwhelm the site of infection, displacing the normal commensal flora [28,29]. A recent study on canine bacterial pneumonia similarly concluded that dogs with pneumonia were more likely to have overgrowth of a single organism, suggesting loss of dominant species associated with health [30], and in human non-cystic fibrosis bronchiectasis, *Pseudomonas aeruginosa* and *Haemophilus influenzae* exhibit strong interspecies competition, leading to competitive exclusion where the dominance of one pathogen coincides with the near-absence of the other [31]. More broadly, severe pneumonia has been associated with significant airway microbiome dysbiosis, characterized by the depletion of commensal bacteria and the expansion of opportunistic pathogens [32]. Collectively, these findings support the role of *B. zoohelcum* as a primary driver of the inflammatory response rather than a secondary colonizer.

To rigorously evaluate alternative etiologies, we also performed metaviromic analysis to examine the potential involvement of viral pathogens. The metaviromic analysis identified numerous DNA viral contigs belonging to the families Anelloviridae, Circoviridae, and Microviridae, but failed to detect any known pathogenic RNA viruses. Given this low abundance, it is unlikely that these viruses alone caused the acute disease. Notably, many of the identified DNA viruses, particularly members of the Anelloviridae family, are typically considered commensals or part of the normal virome of the mammalian respiratory tract [33,34]. Due to the single-sample nature of this study, we cannot exclude the possibility that these viruses contributed to hemorrhagic pneumonia. Future studies with larger sample sizes and experimental models are needed to assess whether these viruses contribute to disease. In addition, although no epidemiological evidence of poisoning was identified and common toxin-producing bacteria such as *Clostridium* spp. were not detected, specific toxicological assays were not performed. Thus, we cannot exclude the possibility that certain bacterial toxins or environmental toxicants (e.g., ricin) contributed to the acute hemorrhagic pneumonia, particularly given that such agents can induce similar pulmonary lesions without characteristic systemic findings [35,36]. Consequently, comprehensive toxicological differential diagnosis should be prioritized when investigating similar cases in the future.

Notably, hemorrhagic pneumonia has been reported in other mammalian species, including mink (caused by *E. coli*) [37], dogs (caused by *Streptococcus equi* subsp. *zooepidemicus*) [38], and Chinese Milu (caused by *Escherichia coli* and *Streptococcus* sp.) [39]. Our findings suggest that *Bergeyella zoohelcum* should be added to this list of pathogens, at least in the context of forest musk deer. Although *B. zoohelcum* is commonly regarded as a commensal of the oral flora of dogs and cats [8], it has also been reported as an opportunistic pathogen in humans, particularly in children [40] and immunocompromised elderly individuals [15], typically following bite wounds. However, its role as a primary cause of fatal hemorrhagic pneumonia in wildlife has remained unrecognized. To date, only two studies have directly linked *B. zoohelcum* to hemorrhagic pneumonia: one on swine [11] and the present study on forest musk deer. Although the genomic determinants of its virulence have yet to be fully characterized—particularly in comparison with the well-defined mechanisms in *S. equinus* [5] or *K. pneumoniae* [6]—the rapid progression to fatality observed in this case unequivocally underscores its high pathogenic capacity. Collectively, these findings challenge the conventional view that *B. zoohelcum* is merely a commensal or an opportunistic pathogen confined to companion animals and humans. We propose that *B. zoohelcum* should be recognized as a pathogen capable of causing severe respiratory disease across diverse mammalian taxa. This expanded pathogenic spectrum has direct implications for veterinary diagnostics in captive wildlife breeding programs and adds a new dimension to the conservation health management of endangered species such as the forest musk deer. Furthermore, given that feral cats and dogs may serve as environmental reservoirs of *B. zoohelcum*, we advocate for the implementation of strict segregation and biosecurity protocols in forest musk deer farms to mitigate the risk of spillover infection.

## 5. Conclusions

In summary, this study provides the first piece of evidence linking *Bergeyella zoohelcum* to fatal hemorrhagic pneumonia in a forest musk deer. The combination of pathology, bacteriology and high-throughput sequencing methods provides a powerful tool for the identification of pathogens. Our findings draw attention to a potentially under-recognized pathogen in captive forest musk deer populations and lay the foundation for future investigations into the epidemiology and pathogenesis of *B. zoohelcum* infection in wildlife.

Study limitations

This study has several limitations inherent to wildlife disease investigation. First, the findings are based on a single fatal case; thus, the prevalence and epidemiological relevance of *B. zoohelcum* in forest musk deer populations remain unknown. Second, despite consistent evidence from bacteriology, histopathology, and viral metagenomics, we could not fulfill Koch’s postulates via experimental infection. Such controlled challenge studies were ethically and logistically infeasible for this endangered species. Third, the transmission route remains unclear. Although *B. zoohelcum* has been isolated from domestic dogs and cats, definitive evidence linking wildlife infections to specific spillover sources is lacking. Hemorrhagic pneumonia episodes occurred shortly after new animal introductions. Transport-related stress may have triggered disease manifestation in carriers. Unfortunately, the lack of traceable provenance records precluded further epidemiological investigation. Fourth, potential sampling biases should be considered. Only postmortem lung tissue from a single case was analyzed, which may have over-represented terminal bacterial proliferation. To address these limitations, in future work, we will focus on two directions. First, we will perform whole-genome sequencing of strain FP and compare it with commensal strains from domestic animals (e.g., cats, dogs) to identify virulence, resistance, and host adaptation determinants. Second, we will conduct longitudinal on-farm screening of forest musk deer and environmental reservoirs (including cats and dogs) to trace transmission routes.

## Figures and Tables

**Table 1:** Summary of de novo assembly results after host sequence subtraction.

Type	Total Base (Mb)	Total Num	Max Len	Min Len	N50	GC (%)
DNA Virome	34.7	30,559	407,966	300	1667	45.86
RNA Virome	1.66	3557	8517	300	461	50.95

**Table 2:** Statistics of viral sequences.

Type	Total Base	Total_Num	Max_Len	Min_Len	N50	GC_Content (%)
DNA	3,832,850	1738	59,511	500	4347	43.79
RNA	5233	7	1313	512	748	49.3

**Table 3:** Statistics of identified viral genome types.

Sample	Total	dsDNA (%)	ssDNA (%)	dsRNA (%)	ssRNA (%)	RT (%)	Unassigned (%)
lung	1738	1156 (66.51%)	346 (19.91)	0	4 (0.23%)	0	232 (13.35)

## Data Availability

The original data presented in the study are openly available in the National Center for Biotechnology Information (NCBI) Sequence Read Archive (SRA) database (Accession Number: PRJNA1470598).

## Supplementary Materials

The following supporting information can be downloaded at: https://www.mdpi.com/article/10.3390/microorganisms14071418/s1, Table S1: Processing and statistics of 16S full-length sequencing data; Table S2: Statistics of raw data and host sequences removal for the DNA virome; Table S3: Summary of sequencing data quality control and removal of ribosomal and host sequences for the RNA virome.

## References

[1] Zhang B.S., Li L.J., Zhu Q., Wang Z., Yuan P., Zhou G.D., Shi W.-J., Chu X.-F., Jiang S., Xie Z.-J. (2020). Co-infection of H9N2 influenza virus and *Pseudomonas aeruginosa* contributes to the development of hemorrhagic pneumonia in mink. Vet. Microbiol..

[2] Li Z., Song K., Du Y., Zhang Z., Fan R., Zheng P., Liu J. (2023). Diagnosis of a Rabbit Hemorrhagic Disease Virus 2 (RHDV2) and the Humoral Immune Protection Effect of VP60 Vaccine. Curr. Issues Mol. Biol..

[3] Yang Q.S., Meng X.X., Xia L., Feng Z.J. (2003). Conservation status and causes of decline of musk deer (*Moschus* spp.) in China. Biol. Conserv..

[4] Han J.J., Feng M.Y., Fang J.Y., Lv D.Y., Wang J.Y. (2024). Isolation and Identification of Pathogenic B acteria Causing Hemorrhagic Pheumonia in *Moschus berezovsky* and Preparation of Bacterial Ghost Vaccine. Chin. J. Vet. Med..

[5] Zhao W., Yu D., Cheng J.G., Wang Y., Yang Z.X., Yao X.P., Luo Y. (2020). Identification and pathogenicity analysis of *Streptococcus equinus FMD1*, a beta-hemolytic strain isolated from forest musk deer lung. J. Vet. Med. Sci..

[6] Yang W., Wang W.Y., Zhao W., Cheng J.G., Wang Y., Yao X.P., Yang Z.-X., Yu D., Luo Y. (2020). Preliminary study on the role of novel LysR family gene kp05372 in *Klebsiella pneumoniae* of forest musk deer. J. Zhejiang Univ. Sci. B.

[7] Hugo C.J., Bruun B., Jooste P.J., Dworkin M., Falkow S., Rosenberg E., Schleifer K.-H., Stackebrandt E. (2006). The Genera *Bergeyella* and *Weeksella*. The Prokaryotes: Volume 7: Proteobacteria: Delta, Epsilon Subclass.

[8] Muramatsu Y., Haraya N., Horie K., Uchida L., Kooriyama T., Suzuki A., Horiuchi M. (2019). *Bergeyella zoohelcum* isolated from oral cavities of therapy dogs. Zoonoses Public Health.

[9] Arechavaleta N.N., Breyer G.M., Siqueira F.M. (2024). *Bergeyella zoohelcum* strain involved in chronic canine rhinitis. An. Acad. Bras. Cienc..

[10] Decostere A., Devriese L.A., Ducatelle R., Haesebrouck F. (2002). *Bergeyella* (*Weeksella*) *zoohelcum* associated with respiratory disease in a cat. Vet. Rec..

[11] Jiang Z., Yaqoob M.U., Siddique A., Guang J., Ed-Dra A., Yue M. (2025). *Bergeyella zoohelcum*: A first case report of its association with respiratory diseases in swine in China. Vet. Res. Commun..

[12] Yi J., Humphries R., Doerr L., Jerris R.C., Westblade L.F. (2016). *Bergeyella zoohelcum* Associated with Abscess and Cellulitis After a Dog Bite. Pediatr. Infect. Dis. J..

[13] Montejo M., Aguirrebengoa K., Ugalde J., Lopez L., Saez Nieto J.A., Hernandez J.L. (2001). *Bergeyella zoohelcum* bacteremia after a dog bite. Clin. Infect. Dis..

[14] Heiberger A., Olcott J., Alzoubaidi M., Free M., Sandeen A. (2025). Ventriculoperitoneal Shunt Infection and Meningitis Due to *Bergeyella zoohelcum* in a Young Child. Pediatr. Infect. Dis. J..

[15] Grams T.R., Kim D.Y., McElvania E. (2023). Closing the Brief Case: *Bergeyella zoohelcum* Bacteremia in an Immunocompromised 69-Year-Old Patient. J. Clin. Microbiol..

[16] Church D.L., Cerutti L., Gürtler A., Griener T., Zelazny A., Emler S. (2020). Performance and Application of 16S rRNA Gene Cycle Sequencing for Routine Identification of Bacteria in the Clinical Microbiology Laboratory. Clin. Microbiol. Rev..

[17] Sudhir K., Glen S., Koichiro T. (2016). MEGA7: Molecular Evolutionary Genetics Analysis Version 7.0 for Bigger Datasets. Mol. Biol. Evol..

[18] Huang Z., Wang Z., Liu Y., Ke C., Feng J., He B., Jiang T. (2024). The links between dietary diversity and RNA virus diversity harbored by the great evening bat (Ia io). Microbiome.

[19] Bolger A.M., Lohse M., Usadel B. (2014). Trimmomatic: A flexible trimmer for Illumina sequence data. Bioinformatics.

[20] Heng L., Richard D. (2009). Fast and accurate short read alignment with Burrows-Wheeler transform. Bioinformatics.

[21] Li D., Luo R., Liu C.M., Leung C.M., Ting H.F., Sadakane K., Yamashita H., Lam T.-W. (2016). MEGAHIT v1.0: A fast and scalable metagenome assembler driven by advanced methodologies and community practices. Methods.

[22] Nayfach S., Camargo A.P., Schulz F., Eloe-Fadrosh E., Roux S., Kyrpides N.C. (2020). CheckV assesses the quality and completeness of metagenome-assembled viral genomes. Nat. Biotechnol..

[23] Guo J., Bolduc B., Zayed A.A., Varsani A., Dominguez Huerta G., Delmont T.O., Pratama A.A., Gazitúa M.C., Vik D., Sullivan M.B. (2021). VirSorter2: A multi-classifier, expert-guided approach to detect diverse DNA and RNA viruses. Microbiome.

[24] Cox P.S., Sharpton T.J., Pollard K.S., DeRisi J.L. (2017). Profile hidden Markov models for the detection of viruses within metagenomic sequence data. PLoS ONE.

[25] Jiang J., Yuan W., Shang J., Shi Y., Yang L., Liu M., Zhu P., Jin T., Sun Y., Yuan L.-H. (2023). Virus classification for viral genomic fragments using PhaGCN2. Brief. Bioinform..

[26] Edgar R.C. (2013). UPARSE: Highly accurate OTU sequences from microbial amplicon reads. Nat. Methods.

[27] Quast C., Pruesse E., Yilmaz P., Gerken J., Schweer T., Yarza P., Peplies J., Glöckner F.O. (2013). The SILVA ribosomal RNA gene database project: Improved data processing and web-based tools. Nucleic Acids Res..

[28] Eskind C.C., Shilts M.H., Boone H.H., Schmitz J.E., Shaver C.M., Satyanarayana G., Das S.R. (2020). Acute infection disrupts the respiratory microbiome of lung transplant recipients. J. Heart Lung Transplant..

[29] Sumner J.T., Hüttelmaier S., Pickens C.I., Moghadam A.A., Abdala-Valencia H., Shen J., Peltekian A., Misharin A.V., Wolfe A.R., Szabo A.L. (2025). Transitions in lung microbiota landscape associate with distinct patterns of pneumonia progression. Cell Host Microbe.

[30] Vientós-Plotts A.I., Ericsson A.C., Rindt H., Reinero C.R. (2019). Respiratory dysbiosis in canine bacterial pneumonia: Standard culture vs. microbiome sequencing. Front. Vet. Sci..

[31] Rogers G.B., van der Giessen C., Serisier D.J. (2015). Predominant pathogen competition and core microbiota divergence in chronic airway infection. ISME J..

[32] Wang Y., Shen Y., Shen J., Bi J., Xu J., Wei T., Wang R., Wu X., Li F., Bai J. (2026). Airway microbiome dysbiosis in severe pneumonia: Metagenomic evidence of pathogen expansion and commensal depletion. Eur. J. Med. Res..

[33] Paietta E.N., Johnston R.A., Randrianarisoa S.F., DeSisto C.M.M., Kraberger S., Martin D., Razanamahenina T.T., Ramboninarimalala A., Velontsara J.-B., Raherinirina T.G. (2026). Host-anellovirus interactions in an island ecosystem: Non-human primates and rodents in Madagascar harbour diverse, rich anellovirus populations. Microb. Genom..

[34] Kaczorowska J., van der Hoek L. (2020). Human anelloviruses: Diverse, omnipresent and commensal members of the virome. FEMS Microbiol. Rev..

[35] Thiel A., Mogel H., Bruggisser J., Baumann A., Wyder M., Stoffel M.H., Summerfield A., Posthaus H. (2017). Effect of *Clostridium perfringens* β-Toxin on Platelets. Toxins.

[36] Bhaskaran M., Didier P.J., Sivasubramani S.K., Doyle L.A., Holley J., Roy C.J. (2014). Pathology of lethal and sublethal doses of aerosolized ricin in rhesus macaques. Toxicol. Pathol..

[37] Lin Y., Wang F., Yan Y., Ma D., Wen S., Wang X., Yang J., Guan Z., Chen H., Ge J. (2025). A virulent *Escherichia coli* O121-B2-ST131 strain causes hemorrhagic pneumonia in mink: Evidence from pathogenicity and animal challenge experiments. BMC Vet. Res..

[38] Simon G., Richard P. (2018). Sudden deaths in greyhounds due to canine haemorrhagic pneumonia. Vet. Rec..

[39] Yan T.L., Zhong Z.Y., Guo Q.Y., Bai J.D., Wang M.H., Wang X.C., Wang X.Y., Cheng Z., Shan Y., Yang C. (2026). Isolation, Identification and Biological Characteristics Analysis of Pathogens Causing Hemorrhagic Pneumonia in New-Born Chinese Milu (*Elaphurus davidianus*). Chin. J. Wildl..

[40] Martínez P., Máttar S. (2006). Zoonotic bacteremia caused by *Bergeyella zoohelcum* in a newborn from Monteria. Infectio.

